# Dietary supplementation of pumpkin seed oil and sunflower oil along with vitamin E improves sperm characteristics and reproductive hormones in roosters

**DOI:** 10.1016/j.psj.2021.101289

**Published:** 2021-05-27

**Authors:** Saied Lotfi, Jafar Fakhraei, Hossein Mansoori Yarahmadi

**Affiliations:** Department of Animal Sciences, Arak Branch, Islamic Azad University, Arak, Iran

**Keywords:** sunflower oil, pumpkin seed oil, reproductive hormones, sperm quality, vitamin E

## Abstract

This study evaluates the effects of pumpkin seed oil and sunflower oil along with vitamin E on the reproductive characteristics of aged roosters. Thirty Ross breeder roosters (45-wk-old) were assigned into 6 groups (5 birds/group) with the following diets: 1) control group (basal diet), 2) basal diet with 2% pumpkin seed oil as omega-3 fatty acid (PSO group), 3) basal diet with 2% sunflower oil as omega-6 fatty acid (SFO group), 4) basal diet with 200 mg/kg vitamin E (Control + vitE group), 5) basal diet and 2% pumpkin seed oil along with 200 mg/kg vitamin E (PSO + vitE group) and 6) basal diet and 2% sunflower oil along with 200 mg/kg vitamin E (SFO + vitE group). Roosters were fed on the experimental diets for 60 d and different characteristics of sperm characteristics including routine semen analysis and several sperm functional tests in every 20 d were examined. Reproductive hormones were also evaluated in 0 d and at the end of the trial. Semen volume and morphology were not affected by any of the diets. The roosters fed with pumpkin seed oil + vitE showed the higher percentage of sperm concentration, total motility, progressive motility, viability and membrane integrity and the lower lipid peroxidation (*P* ≤ 0.05). The group 5 (PSO + vitE) had numerically the lowest sperm with fragmented DNA (DNA Fr^+^) at 0 of the experiment and sperm with non-fragmented DNA (DNA Fr^−^) was lowest in group 6 (SFO + vitE) on da 40 the experiment. Testosterone level was not affected by the experimental diets (*P* > 0.05), however other hormones (LH and FSH) were affected. Based on the results, the supplementation of aged roosters’ diet with pumpkin seed oil + vitE improves reproductive performance which can be an appropriate strategy to preserve the reproductive performance of aged roosters.

## INTRODUCTION

In poultry industry, the reproductive performance of roosters is crucially important. The higher potential capacity of aged breeder roosters will result in the higher reproductive performance of commercial herds (Safari Asl et al., 2018). The broiler breeder roosters encounter an age-related reduction in reproductive performance which will be highlighted at 45 wk of age onward (Abbaspour et al., 2020). Decreased fertility in roosters older than 45 wk is one of the problems of chicken farms. In such roosters, sperm quality is reduced, which can reduce fertility to a level that could not be economically valuable for breeders (Safari Asl et al., 2018). Thus, it is necessary to increase the fertility rate of such roosters, which apart from the health risks of this operation, the return of sperm to the herd will not be done to the desired level. Feeding strategies based on physiological mechanisms can change the physiological conditions of roosters and lead to better fertility.

Polyunsaturated fatty acids (**PUFAs**) are connected to the reproduction functions, metabolism, endocrine and fertilization events (Feng et al., 2015). The roles of omega-3 PUFAs on reproductive performances of roosters have been studied in the recent years (Gulliver et al., 2012). The supplementation of poultry diets with omega-3 sources is necessary since they cannot produce the omega-3 PUFAs due to the lack of specific enzymes in their body (Cerolini et al., 2005). Alagawany et al. (2019), showed that fatty acid supplementation (omega-3 and omega-6), improved semen quality and quantity such as plasma membrane functionality, motility, progressive motility, and viability in roosters. The omega-6 fatty acids are positively related to the sperm motility in chicken also even if present in small proportion (Cerolini et al., 2005). The supplementation of omega-3 from 48 to 58 wk of age is also promising on sperm quality and fertility in turkey breeders (Blesbois et al., 2004). The flaxseed oil (omega-3) along with antioxidant improved the reproductive performance of aged roosters (Pasha Zanussi et al., 2019).

Ezazi et al. (2019), mentioned that sunflower oil (**SF**O; a rich source of PUFAs) and vitamin C improved the reproductive performance in the rams. The other study showed that inclusion of SFO (omega-6) and fish oil (omega-3) improved cryopreservation of rooster semen and positively affected semen parameters post freezing-thawing (Poorvosooghi Gandeshmin et al., 2020). Moreover, supplementation of roosters’ diet with fish oil (omega-3) vs. SFO (omega-6) was more effective on the mentioned factors. Pumpkin seed oil (**PSO**; omega-3 fatty acid source) supplementation prevented changes in plasma lipids and also has shown antiperoxidative properties (Elfiky et al., 2012). The increased sperm count and the decreased DNA fragmentation were observed by supplementing PSO in the human trial (Elfiky et al., 2012). Aghaie et al. (2016), showed that PSO improved semen parameters in adult male Sprague-Dawley rats. Vitamin E is applied to the poultry diet to prevent from the lipid peroxidation caused by PUFAs (Esmaeili et al., 2014). It has been shown that dietary intake of PSO and vitE improved reproductive function of male rats (Hashemi, 2013).

As far as we know, there is no study on the effect of dietary pumpkin oil as a source of omega-3 fatty acids and sunflower seed oil as a source of omega-6 with and without vitamin E on rooster's reproductive performance. Therefore, the present study aims to investigate the effects of the above mentioned on several sperm characteristics such as concentration, motility, viability, morphology, plasma membrane integrity, DNA fragmentation, mitochondrial activity, Apoptosis status, and lipid peroxidation of aged broiler breeder rooster's sperm and also investigates some hormonal changes related to reproduction of aged roosters.

## MATERIALS AND METHODS

### Chemicals

In this study, the chemicals were purchased from Merck (Darmstadt, Germany) and Sigma-Aldrich Company (St. Louis, MO). The Animal Welfare Committee in the Department of Animal Science, Islamic Azad University, Arak Branch, Arak, Iran has approved all experimental procedures.

### Bird's Management and Experimental Design

This study consisted of factorial arrangements (3 × 2) in a randomized complete block design with 5 replications in each treatment. Thirty broiler breeder roosters (Ross 308; age: 45 wk; weight: 4900 ± 210 g) were housed in individual cages (60 *×* 50 *×* 75 cm). The birds were reared under 14 Light: 10 Dark and 21 to 23°C for 10 wk, containing 2 wk of adaptation periods and 8 wk of experimental periods. A standard diet for Ross broiler breeder roosters was fed as the basal diet (Table 1). The experimental diets were 1) basal diet as control group, 2) basal diet with PSO (2%) as n-3 source of fatty acids (PSO group), 3) basal diet with SFO (2%) as n-6 source of fatty acids (SFO group), 4) basal diet with 200 mg/kg vitamin E (Control + vitE group), 5) basal diet and PSO (2%) along with 200 mg/kg vitamin E (PSO + vitE group) and 6) basal diet with SFO (2%) along with 200 mg/kg vitamin E (SFO + vitE group).Water was provided ad libitum and diets were balanced as isoenergetic and isonitrogenous. Table 2 shows the fatty acid compositions of experimental diets, respectively. The analog of vitE source was acetated alpha-tocopherol (Roshd Company, Tehran, Iran), SFO as a source of omega-6 FAs and PSO as a source omega-3 fatty acid (Nina Company, FRICO, Tehran, Iran) were used in the study. They ate their experimental diets from d 0 to d 60 (totally for 60 d). They had a 2 weeks’ adaptation before starting d 0, therefore at d 0, they started to eat the experimental diets and the first semen collection was performed at d 0. The initial quality was assessed on day 0, individually. All the measurements were at days of 0, 20, 40, and 60 of the experiment except for hormonal analysis at d 0 and 60 of the experiment.

**Table 1:** Ingredient and composition of basal diet.

Item	Value
Ingredient (%)	
Corn	60.00
Soybean meal	8.00
Wheat bran	23.35
Sodium bicarbonate	0.10
Salt	0.30
Dicalcium phosphate	1.20
DL- Methionine	0.05
Bentonite	3.50
CaCO3	1.00
Mineral premix 1	0.25
Vitamin premix 2	0.25
Oil	2.00
*Total*	*100*
Calculated composition	
ME 3 (Kcal/Kg)	2708
DM 4	85.73
CP 5 (%)	11.53
Ca (%)	0.70
Nonphytate phosphorous (%)	0.35

1 Supplied per kilogram of diet: Fe (FeSO4•H2O), 90 mg; Mn (MnSO4•H2O), 90 mg; Zn (ZnO), 67.3 mg; Cu (CuSO4•5H2O), 10.9 mg; and Se (Na2SeO3), 0.18 mg.

2 Supplied per kilogram of diet: vitamin A, 15,000 IU; vitamin E, 30 mg; vitamin K3, 4 mg; vitamin D3, 3,000 IU; Riboflavin, 7.5 mg; Pyridoxine, 5.5 mg; vitamin B12, 25 μg; Biotin, 50 μg; Niacin, 50 mg; Calcium pantothenate, 18 mg; and Folic acid, 1.5 mg.

3 ME: Metabolizable energy.

4 DM: Dry matter.

5 CP: Crude protein.

**Table 2:** Fatty acids composition of experimental diets (g/kg of diet).

Fatty acids (%)	Basal diet	Basal diet+VitE	SFO diet	PSO diet
Myristic (C14:0)	ND^1^	ND	ND	1.6
Palmitic (C16:0)	38	38	6	14.8
Stearic (C18:0)	0.2	0.2	0.55	0.9
Arachidic (C20:0)	0.3	0.3	0.4	0.5
*Total SFA*^2^	38.5	38.5	6.95	17.8
Palmitoleic (C16:1)	36	36	5	7.2
Oleic (C18:1 n-9; OA)	72	72	9.5	25.7
Eicosenoic (C20:1)	0.03	0.03	0.3	0.8
Erucic (C22:1)	ND	ND	ND	3.5
*Total MUFA*^3^	10.1	10.08	14.8	37.2
Linoleic (C18:2 n-6; LA)	18.8	18.8	28.1	25.9
Linolenic (C18:3 n-3; LNA)	0.8	0.8	0.8	1.67
Arachidonic (C20:4 n-6; AA)	0.02	0.02	ND	ND
Eicosapentaenoic (C20:5 n-3; EPA)	ND	ND	ND	ND
docosahaexanoic (C22:6 n-3; DHA)	ND	ND	ND	0.44
*Total PUFA*^4^	19.62	19.62	28.90	71.57

1 ND: no detection.

2 SFA (Saturated fatty acids: the sum of C:14, C:16, C:18 and C:20).

3 MUFA (Monounsaturated fatty acids: the sum of C16:1, C18:1, C20:1 and C22:1).

4 PUFA (Polyunsaturated fatty acids: the sum of C18:2, C18:3, C20:5 and C22:6).

### Semen Collection and Assessment of Semen Quality

Semen samples were collected from each bird by the massage in the abdominal section according to semen quality score (sperm concentration × volume) of 2.55 ± 1.25 (mean ± SEM). Samples (semen) were transferred to the laboratory (38°C) within 15 min. The graduated collecting tubes were used for the measurement of semen volume. For sperm concentration, a droplet of semen was placed on the Neubauer hemocytometer after dilution of semen sample (1:200 with distilled water). Motion characteristics of sperm were analyzed using Sperm Class Analyzer software (**SCA**; Version 5.1; Microptic, Barcelona, Spain) as described by Lotfi et al. (2017) and following parameters were measured: total motility (TM, %); progressive motility (PM, %); average path velocity (VAP, μm/s); straight-line velocity (VSL, μm/s); curvilinear velocity (VCL, μm/s); amplitude of lateral head displacement (ALH, μm); beat/cross frequency (BCF, Hz); straightness (STR, %), and linearity (LIN, %).

The evaluation of sperm viability was performed by eosin-nigrosin stain method (Fattah et al., 2017). For this purpose, smears were prepared on a warm slide and the stain was spread on a second slide. Sperm displaying partial or complete purple staining were considered nonviable; only sperm showing strict exclusion of the stain were counted as viable. Viability was assessed by counting 300 sperms under phase-contrast (E200, Nikon, Tokyo, Japan) (× 1,000 magnification; oil immersion). For evaluation of total sperm abnormalities, Hancock's solution was used for sperm dilution (Schäfer and Holzmann, 2000). Then, 10 μL of processed sperm was handled on a slide. The percentage of sperm abnormalities was recorded by counting a total of 300 sperm under a phase-contrast microscope (E200, Nikon, Tokyo, Japan) (× 1,000 magnification; oil immersion). For the evaluation of plasma membrane functionality of sperm Hypo-osmotic swelling test (**HOST**) was used. In brief, mixing 5 µL of semen with a 50 µL hypo-osmotic solution (19.2 mM sodium citrate, 57.6 mM fructose and 100 mum/l) After 30 min incubation, membrane integrity was checked the sperm under a phase-contrast microscope (E200, Nikon, Tokyo, Japan) (× 1,000 magnification; oil immersion) and 300 sperm with swollen and nonswollen tails were recorded (Revell and Mrode, 1994).

### Apoptosis Status and Mitochondrial Activity and Flow Cytometer Procedure

Apoptosis status was identified by a Commercial Kit (IQP, Groningen, the Netherlands). The calcium buffer washed the sperm samples and readjusted the concentration to 1 × 10^6^ sperm/mL, followed by the addition of 10 µL of Annexin V-FITC (0.01 mg/mL) to 100 mL of sperm suspension. This was then incubated for 20 min on ice. Next, 10 mL of propidium iodide (**PI**; 1 mg/ mL) was added to the sperm suspension and incubated for at least 10 min on ice. Then, the suspension was evaluated using a flow cytometer. The sperm samples were classified into 4 groups: 1) viable non-apoptotic cells; 2) early apoptotic cells;3) late apoptotic cells, 4) necrotic cells death. The late apoptotic and necrotic cells were categorized as dead cells (Lotfi et al., 2017). Semen samples were stained with JC-1 stain and assessed for their mitochondrial activity using flow cytometry. Briefly, Stock solutions of 0.13 mg JC-1 (T3168, Molecular Probes, Eugene, OR) were prepared in DMSO and maintained at 37°C while shielded from light. 1.0 µL of the JC-1 solution (2.0 µm/L) was added to 500 µL of the PBS-diluted semen sample (50 × 10^6^ sperm/mL) and incubated for 20 min at 37°C in the dark. The samples thus obtained were then centrifuged at 200 *g* for 10 min before the sperm pellets were resuspended in 500 µL PBS buffer samples for evaluation by flow cytometer (Najafi et al., 2021). All analyses were carried out by one experienced technician blinded to the study. For flow cytometer procedure, all fluorescence signals of labeled spermatozoa were analyzed by a FACS Calibur flow cytometer (Becton Dickinson, San Jose, CA) equipped with standard. Approximately 10,000 sperm cell events were examined with a flow rate of <200 events/second. The analysis of flow cytometry data was performed using FlowJo software (Treestar, Inc., San Carlos, CA).

### Malondialdehyde Formation, Sperm DNA Fragmentation

Malondialdehyde (**MDA**) concentrations of semen samples were assessed as an index of lipid peroxidation were measured by using the thiobarbituric-acid reaction (Masoudi et al., 2017). Briefly, 1 mL of the diluted semen sample (250 × 10^6^ sperm/mL) was mixed with 1 mL of cold 20% (w/v) tricholoro acetic acid to precipitate protein. The precipitate was pelleted by centrifuging (960 g for 15 min), and 1 mL of the supernatant was incubated with 1 mL of 0.67% (w/v) thiobarbituric acid in a boiling water bath at 100°C for 10 min. After cooling, the absorbance was determined using a spectrophotometer (UV-1200, Shima-dzu, Japan) at 532 nm. All MDA concentrations were expressed as nmol/ml.

Sperm DNA fragmentation (**SDF**) was evaluated by using the SDF kit (Hooshmand Fanavar Tehran Co., Iran) as described by Oghbaei et al. (2020). The SDF Kit was designed based on the sperm chromatin dispersion technique that normal spermatozoa create complete and massive halos formed by loops of DNA at the head of the sperm, which are not present in those with damaged DNA. All analyses were carried out by one experienced technician blinded to the study.

### Assessment of Reproductive Hormones

The blood samples were obtained at 0 and 60 days of the experiment. In brief, plasma was obtained by centrifuging at 3,000 rpm for 10 min at 4°C and stored at −20°C. The concentrations of luteinizing hormone (**LH**), and follicle-stimulating hormone (**FSH**) were measured using a chicken ELISA kit (Crystal Day, Shanghai, China), according to the manufacturer's instruction. Testosterone was measured using an ELISA kit (Monobind Inc., Costa Mesa, CA). Intra-assay coefficients of variation and sensitivity of the testosterone assay were 6.08% and 0.0576 ng/mL, respectively.

### Statistical Analysis

The design model in this study is completely randomized with 6 treatments, which is obtained by combining the diet with PSO, SFO, and vitamin E in 5 replications. The statistical model was as follows: Y_ij_ = µ+ A_ij_+∑ij, Where, Y_ij_ is observation, µ is the average, Aij is the effects of treatments and ∑ij is the experimental error.

The data were statistically analyzed using SAS software. All data are presented as least squares means+SEM. Each individual rooster was considered as an experimental unit in all statistical analyses. Analysis and means were compared using Tukey test. In order for sperm characteristics to be affected by the standard (control) diet fed for 2 wk, sperm characteristics were considered as an accompanying variable at the beginning of the experiment and data on those traits were analyzed by analysis of covariance (**ANCOVA**) and decomposed by the following model: Yij = β0+ β1Xij+ τi+εij, Where, Yij is the observation, β0 is intercept, B1 is regression, Xij is covariate variable, τi is treatment effect and εij is experimental error. In all statistical analyses, the significance level was set at *P <* 0.05.

## RESULTS

### Sperm Quality and Quantity Parameters

**Figure 1:**
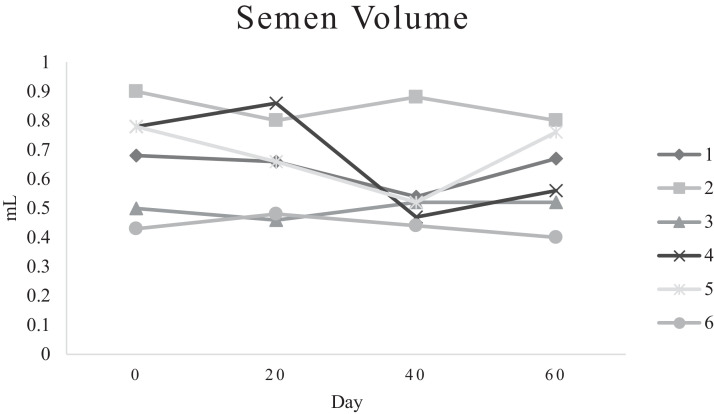
Means of semen volume in aged roosters fed diets during 60 days’ experiment. Roosters received 6 types of treatments: 1) basal diet as control group (Control), 2) basal diet supplemented with 2% pumpkin seed oil (PSO group), 3) basal diet supplemented with 2% sunflower oil (SFO group), 4) basal diet supplemented with 200 mg/kg vitamin E (CTRL + vitE group), 5) basal diet supplemented and 2% pumpkin seed oil + 200 mg/kg vitamin E (PSO + vitE group) and 6) basal diet supplemented with 2% sunflower oil + 200 mg/kg vitamin E (SFO + vitE group) (n = 6 birds per each treatment). ^a-d^ Different letters within each day show significant differences among the groups (*P* ≤ 0.05). Abbreviations: PSO, pumpkin seed oil; SFO, sunflower oil; vitE, vitamin E.

**Figure 2:**
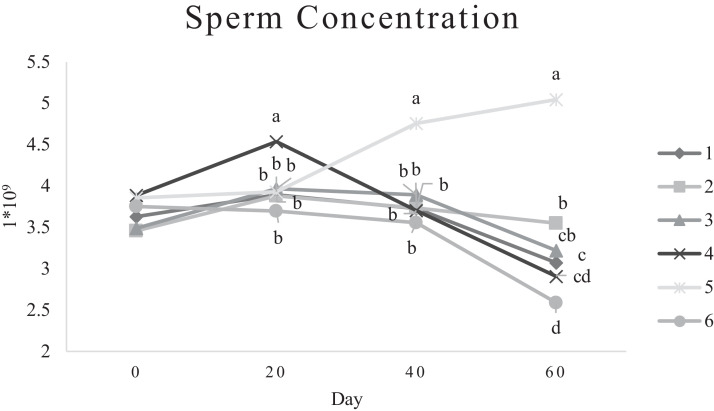
Means of sperm concentration in aged roosters fed diets during 60 days’ experiment. Roosters received 6 types of treatments: 1) basal diet as control group (Control), 2) basal diet supplemented with 2% pumpkin seed oil (PSO group), 3) basal diet supplemented with 2% sunflower oil (SFO group), 4) basal diet supplemented with 200 mg/kg vitamin E (CTRL + vitE group), 5) basal diet supplemented and 2% pumpkin seed oil + 200 mg/kg vitamin E (PSO + vitE group) and 6) basal diet supplemented with 2% sunflower oil + 200 mg/kg vitamin E (SFO + vitE group) (n = 6 birds per each treatment). ^a-d^ Different letters within each day show significant differences among the groups (*P* ≤ 0.05). Abbreviations: PSO, pumpkin seed oil; SFO, sunflower oil; vitE, vitamin E.

**Figure 3:**
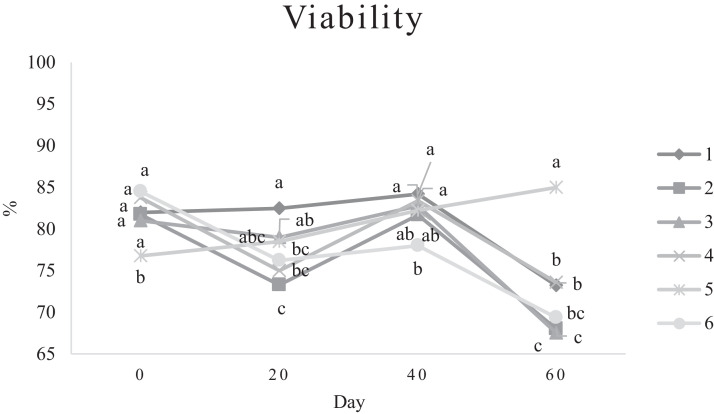
Means of sperm viability in aged roosters fed diets during 60 days’ experiment. Roosters received 6 types of treatments: 1) basal diet as control group (Control), 2) basal diet supplemented with 2% pumpkin seed oil (PSO group), 3) basal diet supplemented with 2% sunflower oil (SFO group), 4) basal diet supplemented with 200 mg/kg vitamin E (CTRL + vitE group), 5) basal diet supplemented and 2% pumpkin seed oil + 200 mg/kg vitamin E (PSO + vitE group) and 6) basal diet supplemented with 2% sunflower oil + 200 mg/kg vitamin E (SFO + vitE group) (n = 6 birds per each treatment). ^a-d^ Different letters within each day show significant differences among the groups (*P* ≤ 0.05). Abbreviations: PSO, pumpkin seed oil; SFO, sunflower oil; vitE, vitamin E.

**Figure 4:**
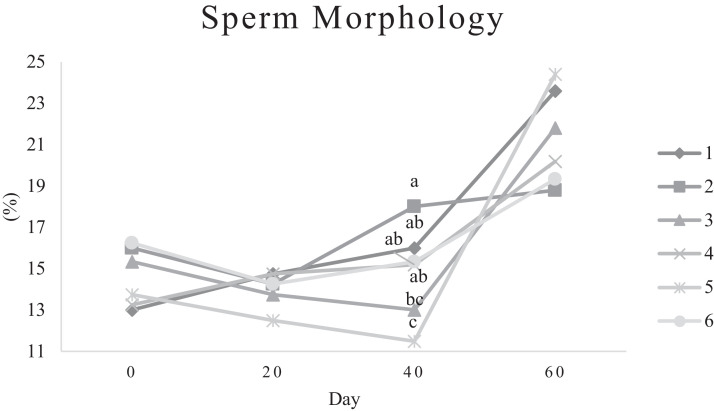
Means of sperm morphology in aged roosters fed diets during 60 days’ experiment. Roosters received 6 types of treatments: 1) basal diet as control group (Control), 2) basal diet supplemented with 2% pumpkin seed oil (PSO group), 3) basal diet supplemented with 2% sunflower oil (SFO group), 4) basal diet supplemented with 200 mg/kg vitamin E (CTRL + vitE group), 5) basal diet supplemented and 2% pumpkin seed oil + 200 mg/kg vitamin E (PSO + vitE group) and 6) basal diet supplemented with 2% sunflower oil + 200 mg/kg vitamin E (SFO + vitE group) (n = 6 birds per each treatment). ^a-d^ Different letters within each day show significant differences among the groups (*P* ≤ 0.05). Abbreviations: PSO, pumpkin seed oil; SFO, sunflower oil; vitE, vitamin E.

**Figure 5:**
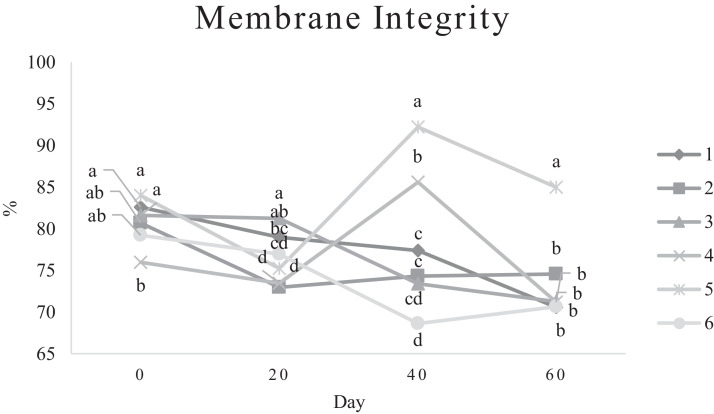
Means of sperm membrane integrity in aged roosters fed diets during 60 days’ experiment. Roosters received 6 types of treatments: 1) basal diet as control group (Control), 2) basal diet supplemented with 2% pumpkin seed oil (PSO group), 3) basal diet supplemented with 2% sunflower oil (SFO group), 4) basal diet supplemented with 200 mg/kg vitamin E (CTRL+ vitE group), 5) basal diet supplemented and 2% pumpkin seed oil + 200 mg/kg vitamin E (PSO + vitE group) and 6) basal diet supplemented with 2% sunflower oil + 200 mg/kg vitamin E (SFO + vitE group) (n = 6 birds per each treatment). ^a-d^ Different letters within each day show significant differences among the groups (*P* ≤ 0.05). Abbreviations: PSO, pumpkin seed oil; SFO, sunflower oil; vitE, vitamin E.

**Figure 6:**
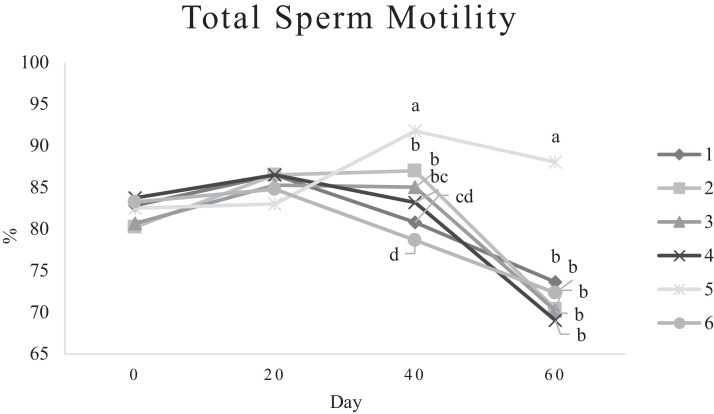
Means of sperm motility in aged roosters fed diets during 60 days’ experiment. Roosters received 6 types of treatments: 1) basal diet as control group (Control), 2) basal diet supplemented with 2% pumpkin seed oil (PSO group), 3) basal diet supplemented with 2% sunflower oil (SFO group), 4) basal diet supplemented with 200 mg/kg vitamin E (CTRL + vitE group), 5) basal diet supplemented and 2% pumpkin seed oil + 200 mg/kg vitamin E (PSO + vitE group) and 6) basal diet supplemented with 2% sunflower oil + 200 mg/kg vitamin E (SFO + vitE group) (n = 6 birds per each treatment). ^a-d^ Different letters within each day show significant differences among the groups (*P* ≤ 0.05). Abbreviations: PSO, pumpkin seed oil; SFO, sunflower oil; vitE, vitamin E.

**Figure 7:**
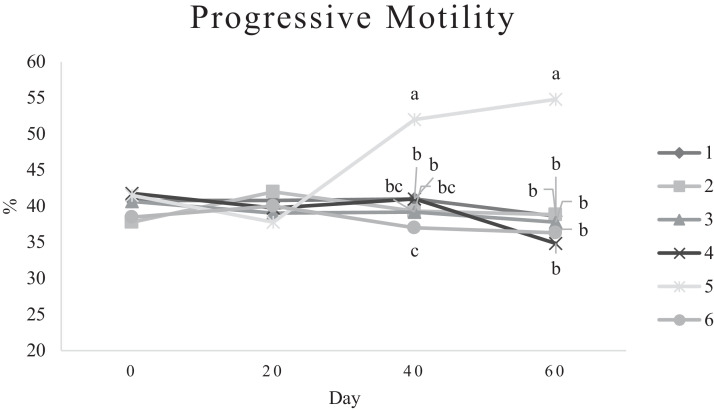
Means of sperm progressive motility in aged roosters fed diets during 60 days’ experiment. Roosters received 6 types of treatments: 1) basal diet as control group (Control), 2) basal diet supplemented with 2% pumpkin seed oil (PSO group), 3) basal diet supplemented with 2% sunflower oil (SFO group), 4) basal diet supplemented with 200 mg/kg vitamin E (CTRL + vitE group), 5) basal diet supplemented and 2% pumpkin seed oil + 200 mg/kg vitamin E (PSO + vitE group) and 6) basal diet supplemented with 2% sunflower oil + 200 mg/kg vitamin E (SFO + vitE group) (n = 6 birds per each treatment). ^a-d^ Different letters within each day show significant differences among the groups (*P* ≤ 0.05).Abbreviations: PSO, pumpkin seed oil; SFO, sunflower oil; vitE, vitamin E.

**Figure 8:**
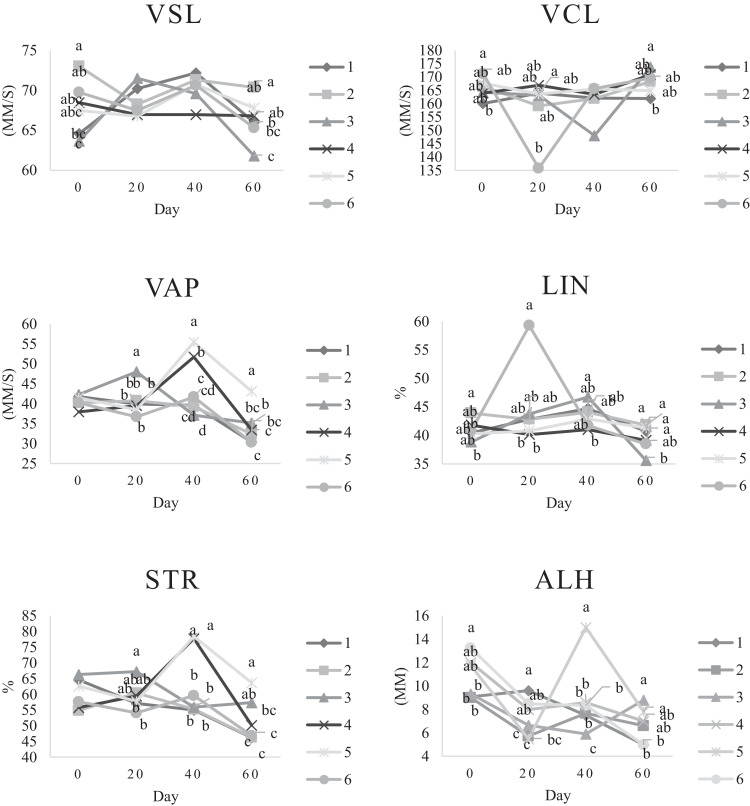
Means of malondialdehyde of sperm in aged roosters fed diets during 60 days’ experiment. Roosters received 6 types of treatments: 1) basal diet as control group (Control), 2) basal diet supplemented with 2% pumpkin seed oil (PSO group), 3) basal diet supplemented with 2% sunflower oil (SFO group), 4) basal diet supplemented with 200 mg/kg vitamin E (CTRL + vitE group), 5) basal diet supplemented and 2% pumpkin seed oil + 200 mg/kg vitamin E (PSO + vitE group) and 6) basal diet supplemented with 2% sunflower oil + 200 mg/kg vitamin E (SFO + vitE group) (n = 6 birds per each treatment). ^a-d^ Different letters within each day show significant differences among the groups (*P* ≤ 0.05).Abbreviations: PSO, pumpkin seed oil; SFO, sunflower oil; vitE, vitamin E.

**Figure 9:**
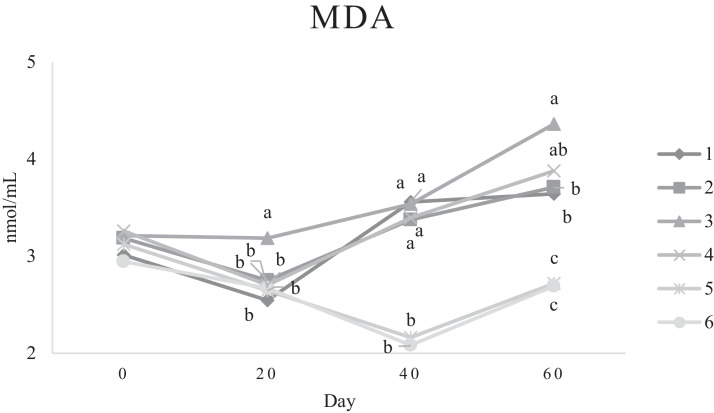
Means of malondialdehyde of sperm in aged roosters fed diets during 60 days’ experiment. Roosters received 6 types of treatments: 1) basal diet as control group (Control), 2) basal diet supplemented with 2% pumpkin seed oil (PSO group), 3) basal diet supplemented with 2% sunflower oil (SFO group), 4) basal diet supplemented with 200 mg/kg vitamin E (CTRL + vitE group), 5) basal diet supplemented and 2% pumpkin seed oil + 200 mg/kg vitamin E (PSO + vitE group) and 6) basal diet supplemented with 2% sunflower oil + 200 mg/kg vitamin E (SFO + vitE group) (n = 6 birds per each treatment). ^a-d^ Different letters within each day show significant differences among the groups (*P* ≤ 0.05).Abbreviations: PSO, pumpkin seed oil; SFO, sunflower oil; vitE, vitamin E.

**Figure 10:**
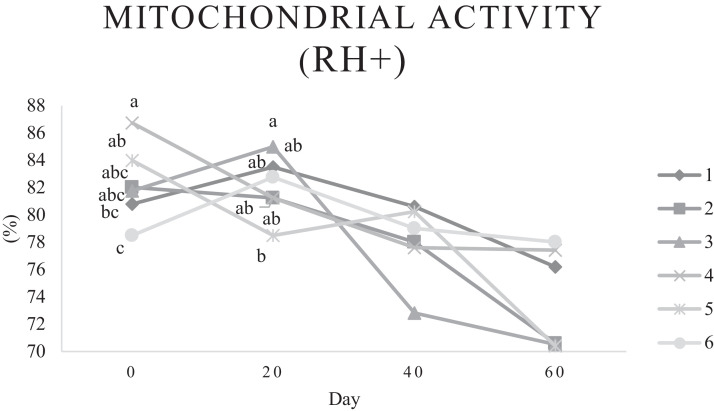
Means of mitochondrial activity in aged roosters fed diets during 60 days’ experiment. Roosters received 6 types of treatments: 1) basal diet as control group (Control), 2) basal diet supplemented with 2% pumpkin seed oil (PSO group), 3) basal diet supplemented with 2% sunflower oil (SFO group), 4) basal diet supplemented with 200 mg/kg vitamin E (CTRL + vitE group), 5) basal diet supplemented and 2% pumpkin seed oil + 200 mg/kg vitamin E (PSO + vitE group) and 6) basal diet supplemented with 2% sunflower oil + 200 mg/kg vitamin E (SFO + vitE group) (n = 6 birds per each treatment). ^a-d^ Different letters within each day show significant differences among the groups (*P* ≤ 0.05).Abbreviations: PSO, pumpkin seed oil; SFO, sunflower oil; vitE, vitamin E.

**Figure 11:**
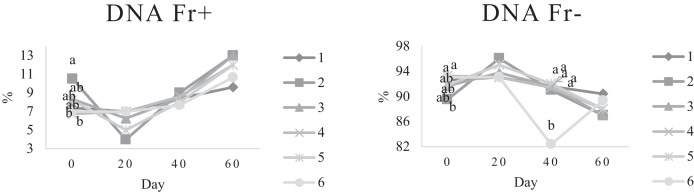
Means of DNA fragmentation of sperm in aged roosters fed diets during 60 days’ experiment. Roosters received 6 types of treatments: 1) basal diet as control group (Control), 2) basal diet supplemented with 2% pumpkin seed oil (PSO group), 3) basal diet supplemented with 2% sunflower oil (SFO group), 4) basal diet supplemented with 200 mg/kg vitamin E (CTRL + vitE group), 5) basal diet supplemented and 2% pumpkin seed oil + 200 mg/kg vitamin E (PSO + vitE group) and 6) basal diet supplemented with 2% sunflower oil + 200 mg/kg vitamin E (SFO + vitE group) (n = 6 birds per each treatment). ^a-d^ Different letters within each day show significant differences among the groups (*P* ≤ 0.05). Abbreviations: PSO, pumpkin seed oil; SFO, sunflower oil; vitE, vitamin E.

**Figure 12:**
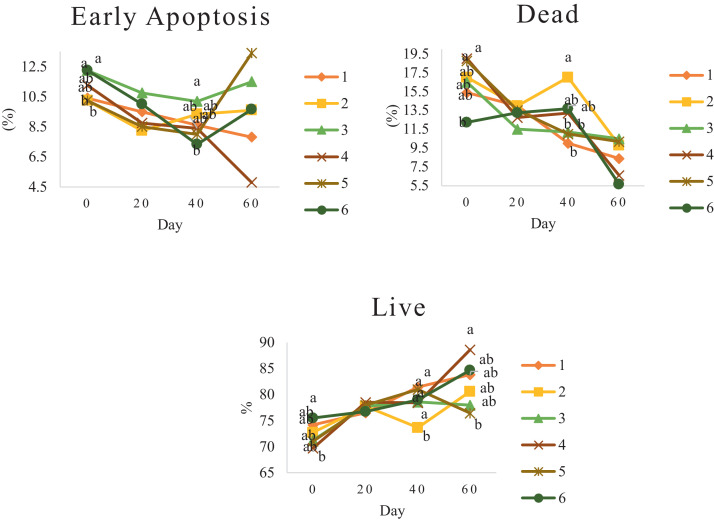
Means of apoptotic status in aged roosters fed diets during 60 days’ experiment. Roosters received 6 types of treatments: 1) basal diet as control group (Control), 2) basal diet supplemented with 2% pumpkin seed oil (PSO group), 3) basal diet supplemented with 2% sunflower oil (SFO group), 4) basal diet supplemented with 200 mg/kg vitamin E (CTRL+vitE group), 5) basal diet supplemented and 2% pumpkin seed oil + 200 mg/kg vitamin E (PSO+vitE group) and 6) basal diet supplemented with 2% sunflower oil + 200 mg/kg vitamin E (SFO+vitE group) (n = 6 birds per each treatment). ^a-d^ Different letters within each day show significant differences among the groups (*P* ≤ 0.05). Abbreviations: PSO, pumpkin seed oil; SFO, sunflower oil; vitE, vitamin E.

Characteristics of sperm appeared to be different at different times of experiment (Table 3, Table 4, Table 5, Figure 1, Figure 2, Figure 3, Figure 4, Figure 5, Figure 6, Figure 7, Figure 8, Figure 9, Figure 10, Figure 11, Figure 12). However, the semen volume and sperm morphology (except at 40 d) were not affected by the experimental diets (Table 3, Figure 1 and Figure 4). In terms of sperm concentration, the group 5 (PSO + vitE group) showed the greatest value as compared with others in d 40 and 60 (Table 3, Figure 2). Viability of sperm were significantly different among the treatments from 0 d until the day 60th. On d 60th, the group 5 (PSO + vitE group) had the greatest value of sperm viability (Table 3, Figure 3). In terms of morphology, none of the treatment groups showed a significant difference on days of 0, 20, and 60, however, on d 40, the group 5 (PSO + vitE) had the least value (Table 3, Figure 4). In terms of plasma membrane integrity, there was a significant difference (*P* < 0.05) among the treatments, however, the group 5 (PSO + vitE) had the greatest membrane integrity on 1, 40, and 60 as compared with others (Table 3, Figure 5).

**Table 3:** Sperm characteristics of roosters fed with fatty acid sources with or without vitamin E.

		Treatments^1^							
Parameters	Days	1 (CTR group)	2 (PSO group)	3 (SFO group)	4 (CTR+vitE group)	5 (PSO+vitE group)	6 (SFO+vitE group)	SEM	*P*-value
Volume (mL)									
	0	0.68	0.9	0.5	0.78	0.78	0.43	0.07	0.28
	20	0.66	0.8	0.46	0.86	0.66	0.48	0.07	0.53
	40	0.54	0.88	0.52	0.47	0.52	0.44	0.06	0.27
	60	0.67	0.8	0.52	0.56	0.76	0.4	0.06	0.29
Concentration (1 × 10^9^)									
	0	3.63	3.46	3.49	3.89	3.86	3.75	0.06	0.17
	20	3.89 ^b^	3.88 ^b^	3.97 ^b^	4.54 ^a^	3.93 ^b^	3.70 ^b^	0.07	0.01
	40	3.73 ^b^	3.74 ^b^	3.89 ^b^	3.70 ^b^	4.76 ^a^	3.55 ^b^	0.1	0.0007
	60	3.07 ^c^	3.55 ^b^	3.22 ^bc^	2.90 ^cd^	5.05 ^a^	2.59 ^d^	0.16	<0.0001
Viability (%)									
	0	82.00 ^a^	81.75 ^a^	81.00 ^a^	83.75 ^a^	76.75 ^b^	84.50 ^a^	0.64	0.002
	20	82.50 ^a^	73.25 ^c^	79.00 ^ab^	75.00 ^bc^	78.50 ^abc^	76.25 ^bc^	0.86	0.02
	40	84.20 ^a^	81.67 ^ab^	82.80 ^a^	83.40 ^a^	82.25 ^ab^	78.00 ^b^	0.63	0.06
	60	73.20 ^b^	68.00 ^c^	67.50 ^c^	73.60 ^b^	85.00 ^a^	69.33 ^bc^	1.25	<0.0001
Morphology (%)									
	0	13	16	15.33	13.25	13.75	16.25	0.51	0.25
	20	14.75	14.25	13.75	14.75	12.5	14.25	0.41	0.66
	40	16.0 ^ab^	18.0 ^a^	13.0 ^bc^	15.20 ^ab^	11.50 ^c^	15.33 ^ab^	0.54	0.002
	60	23.6	18.8	21.8	20.2	24.4	19.33	0.85	0.31
Membrane Integrity (%)									
	0	82.60 ^a^	80.75 ^ab^	81.67 ^a^	76.00 ^b^	84.00 ^a^	79.25 ^ab^	0.77	0.03
	20	79.00 ^ab^	73.00 ^d^	81.25 ^a^	73.50 ^d^	75.25 ^cd^	77.00 ^bc^	0.69	0.0001
	40	77.40 ^c^	74.33 ^c^	73.40 ^cd^	85.60 ^b^	92.25 ^a^	68.67 ^d^	1.6	<0.0001
	60	70.60 ^b^	74.60 ^b^	71.25 ^b^	71.20 ^b^	85.00 ^a^	70.67 ^b^	1.09	<0.0001

Data are presented as means ± SEM (n = 5 birds per each treatment).

Sperm Parameters: Morphology (Hancock solution), viability (Eosin-Nigrosin), plasma membrane integrity (Hypo-osmotic swelling test) were assessed.

Abbreviations: PSO, pumpkin seed oil; SFO, sunflower oil; vitE, vitamin E.

^a-b^Different letters within the same row show significant differences among the groups (*P* < 0.05).

1 Roosters received 6 types of treatments: 1) basal diet as control group (Control), 2) basal diet supplemented with 2% pumpkin seed oil as n-3 source of fatty acids (PSO group), 3) basal diet supplemented with 2% sunflower oil as n-6 source of fatty acids (SFO group), 4) basal diet supplemented with 200 mg/kg vitamin E (CTRL + vitE group), 5) basal diet supplemented and 2% pumpkin seed oil along with 200 mg/kg vitamin E (PSO + vitE group) and 6) basal diet supplemented with 2% sunflower oil along with 200 mg/kg vitamin E (SFO + vitE group).

**Table 4:** Sperm characteristics of roosters fed with fatty acid sources with or without vitamin E.

		Treatments^1^							
Parameters	Days	1 (CTR group)	2 (PSO group)	3 (SFO group)	4 (CTR+vitE group)	5 (PSO+vitE group)	6 (SFO+vitE group)	SEM	*P*-value
Total motility (TM, %)									
	0	82.8	80.25	80.67	83.75	82.5	83.25	0.58	0.45
	20	86.5	86.5	85.25	86.5	83	84.75	0.49	0.24
	40	80.80 ^cd^	87.00 ^b^	85.00 ^b^	83.20 ^bc^	91.75 ^a^	78.67 ^d^	0.94	<0.0001
	60	73.60 ^b^	70.40 ^b^	70.20 ^b^	69.00 ^b^	88.00 ^a^	72.33 ^b^	1.36	<0.0001
Progressive motility (PM, %)									
	0	40.8	37.75	40.67	41.75	41.5	38.5	0.64	0.38
	20	40.75	42	39	39.75	37.75	40	0.55	0.33
	40	41.00 ^b^	39.33 ^bc^	39.20 ^bc^	41.00 ^b^	52.00 ^a^	37.00 ^c^	1	<0.0001
	60	38.60 ^b^	38.80 ^b^	37.80 ^b^	34.80 ^b^	54.80 ^a^	36.33 ^b^	1.35	<0.0001
VSL (µm/s)^2^									
	0	64.60 ^bc^	73.00 ^a^	63.67 ^c^	68.50 ^abc^	67.50 ^abc^	69.75 ^ab^	0.93	0.012
	20	70.25	68.25	71.5	67	66.75	67.5	0.69	0.653
	40	72.2	71.33	69.6	67	70.75	70.67	0.7	0.3462
	60	66.00 ^b^	70.40 ^a^	61.80 ^c^	66.80 ^ab^	67.80 ^ab^	65.33 ^bc^	0.99	0.0059
VAP (µm/s)^3^									
	0	41.8	40.25	42.33	38	41.5	40.25	0.99	0.8616
	20	40.00 ^b^	40.75 ^b^	48.00 ^a^	39.50 ^b^	38.50 ^b^	36.75 ^b^	0.97	0.0076
	40	39.60 ^cd^	39.33 ^cd^	37.20 ^d^	51.80 ^b^	55.50 ^a^	41.67 ^c^	1.33	<0.0001
	60	30.40 ^c^	32.40 ^bc^	35.20 ^b^	33.40 ^bc^	43.20 ^a^	30.33 ^c^	0.97	<0.0001
LIN (%)^4^									
	0	40.42 ^ab^	43.89 ^a^	38.81 ^b^	41.78 ^ab^	40.27 ^ab^	40.80 ^ab^	0.56	0.1542
	20	42.81 ^ab^	42.80 ^ab^	43.78 ^ab^	40.13 ^b^	40.81 ^b^	59.30 ^a^	2.32	0.1503
	40	44.73 ^ab^	44.08 ^ab^	46.75 ^a^	41.04 ^b^	42.91 ^ab^	41.78 ^ab^	0.69	0.1663
	60	40.76 ^a^	41.84 ^a^	35.57 ^b^	39.11 ^a^	41.31 ^a^	38.50 ^ab^	0.56	0.0042
VCL (µm/s)^5^									
	0	160.20 ^b^	166.50 ^ab^	164.33 ^ab^	164.00 ^ab^	167.75 ^ab^	171.25 ^a^	1.12	0.08
	20	164.00 ^ab^	159.25 ^ab^	163.25 ^ab^	167.00 ^a^	163.75 ^ab^	135.75 ^b^	3.81	0.17
	40	162.2	162	148	163.6	165.5	165.67	2.3	0.22
	60	162.00 ^b^	168.40 ^ab^	173.80 ^a^	171.00 ^ab^	165.00 ^ab^	169.67 ^ab^	1.38	0.16
STR (%)^6^									
	0	64.69	54.96	66.34	55.84	62.65	57.72	1.74	0.28
	20	57.15 ^b^	60.39 ^ab^	67.22 ^a^	59.50 ^ab^	57.86 ^ab^	54.18 ^b^	1.36	0.1
	40	54.98 ^b^	55.32 ^b^	56.00 ^b^	77.71 ^a^	78.45 ^a^	59.63 ^b^	2.09	<0.0001
	60	46.17 ^c^	46.32 ^c^	57.39 ^ab^	50.13 ^bc^	63.71 ^a^	46.88 ^c^	1.61	0.0005
ALH (µm)^7^									
	0	9.10 ^b^	9.00 ^b^	9.33 ^b^	12.00 ^ab^	11.75 ^ab^	13.25 ^a^	0.53	0.06
	20	9.63 ^a^	5.75 ^c^	6.65 ^bc^	8.00 ^ab^	5.50 ^c^	8.50 ^ab^	0.38	0.001
	40	7.50 ^b^	7.67 ^b^	5.90 ^c^	8.60 ^b^	15.00 ^a^	8.33 ^b^	0.57	<0.0001
	60	5.00 ^b^	6.60 ^ab^	8.80 ^a^	7.00 ^ab^	7.80 ^a^	5.00 ^b^	0.38	0.01

Data are presented as means ± SEM (n = 5 birds per each treatment).

Sperm Parameters: Total motility, progressive motility and sperm kinematic parameters (Sperm Class Analyzer Soft.) were assessed.

Abbreviations: PSO, pumpkin seed oil; SFO, sunflower oil; vitE, vitamin E.

^a-b^ Different letters within the same row show significant differences among the groups (*P* < 0.05).

1 Roosters received 6 types of treatments: 1) basal diet as control group (Control), 2) basal diet supplemented with 2% pumpkin seed oil as n-3 source of fatty acids (PSO group), 3) basal diet supplemented with 2% sunflower oil as n-6 source of fatty acids (SFO group), 4) basal diet supplemented with 200 mg/kg vitamin E (CTRL + vitE group), 5) basal diet supplemented and 2% pumpkin seed oil along with 200 mg/kg vitamin E (PSO + vitE group) and 6) basal diet supplemented with 2% sunflower oil along with 200 mg/kg vitamin E (SFO + vitE group).

2 VSL: average velocity measured in a straight line from the beginning to the end of track.

3 VAP: path velocity of the smoothed cell path.

4 LIN: average value of the ratio VSL/VCL; measured the departure of the cell track from a straight line.

5 VCL: average velocity measured over the actual point to point track followed by the cell.

6 STR: average value of the ratio VSL/VAP; measured the departure of the cell path from a straight line.

7 ALH: amplitude of lateral head displacement corresponding to the mean width of the head oscillation as the sperm swam.

**Table 5:** Sperm characteristics of roosters fed with fatty acid sources with or without vitamin E.

		Treatments^1^							
Parameters	Days	1 (CTR group)	2 (PSO group)	3 (SFO group)	4 (CTR+vitE group)	5 (PSO+vitE group)	6 (SFO+vitE group)	SEM	*P*-value
MDA (nmol/mL)									
	0	3.01	3.19	3.21	3.26	3.12	2.94	0.07	0.79
	20	2.55 ^b^	2.75 ^b^	3.19 ^a^	2.70 ^b^	2.65 ^b^	2.67 ^b^	0.06	0.03
	40	3.56 ^a^	3.37 ^a^	3.54 ^a^	3.39 ^a^	2.16 ^b^	2.08 ^b^	0.14	<0.0001
	60	3.64 ^b^	3.71 ^b^	4.36 ^a^	3.88 ^ab^	2.72 ^c^	2.69 ^c^	0.13	<0.0001
Mitochondrial activity (%)									
	0	80.80 ^bc^	82.00 ^abc^	81.75 ^abc^	86.75 ^a^	84.00 ^ab^	78.50 ^c^	0.03	0.78
	20	83.50 ^ab^	81.25 ^ab^	85.00 ^a^	81.25 ^ab^	78.50 ^b^	82.75 ^ab^	0.15	0.73
	40	80.6	78	72.8	77.6	80.25	79	0.52	1.23
	60	76.2	70.6	70.5	77.4	70.4	78	0.69	1.86
DNA Fr+ (%)									
	0	7.40 ^ab^	10.50 ^a^	8.33 ^ab^	6.75 ^b^	8.25 ^ab^	7.00 ^b^	0.46	0.18
	20	7	4	6.25	7	5	7	0.42	0.16
	40	8.4	9	8.4	8.6	8	7.67	0.31	0.89
	60	9.6	13	13	12	12	10.67	0.54	0.42
DNA Fr^−^ (%)									
	0	92.60 ^ab^	89.50 ^b^	91.67 ^ab^	93.25 ^a^	91.75 ^ab^	93.00 ^a^	0.46	0.18
	20	93	96	93.75	93	95	93	0.42	0.16
	40	91.60 ^a^	91.00 ^a^	91.60 ^a^	91.40 ^a^	92.00 ^a^	82.33 ^b^	1.04	0.03
	60	90.4	87	87	88	88	89.33	0.54	0.42
Apoptosis status									
Live (%)									
	0	74.20 ^ab^	72.75 ^ab^	71.25 ^ab^	69.75 ^b^	71.00 ^ab^	75.50 ^a^	0.72	0.17
	20	76.5	77.75	77.75	78.5	78	76.75	0.6	0.94
	40	81.40 ^a^	73.67 ^b^	78.60 ^a^	78.40 ^a^	81.00 ^a^	79.00 ^a^	0.63	0.001
	60	83.80 ^ab^	80.60 ^ab^	78.00 ^ab^	88.60 ^a^	76.40 ^b^	84.67 ^ab^	1.48	0.15
Early apoptotic (%)									
	0	10.40 ^ab^	10.25 ^b^	12.25 ^a^	11.25 ^ab^	10.25 ^b^	12.25 ^a^	0.28	0.05
	20	9.5	8.25	10.75	8.75	8.5	10	0.38	0.38
	40	8.60 ^ab^	9.33 ^ab^	10.20 ^a^	8.40 ^ab^	8.00 ^ab^	7.33 ^b^	0.36	0.24
	60	7.8	9.6	11.5	4.8	13.4	9.67	1.12	0.33
Necrotic cells death (%)									
	0	15.40 ^ab^	17.00 ^ab^	16.50 ^ab^	19.00 ^a^	18.75 ^a^	12.25 ^b^	0.82	0.18
	20	14	14	11.5	12.75	13.5	13.25	0.71	0.93
	40	10.00 ^b^	17.00 ^a^	11.20 ^b^	13.20 ^ab^	11.00 ^b^	13.67 ^ab^	0.69	0.03
	60	8.4	9.8	10.5	6.6	10.2	5.67	0.65	0.15

Data are presented as means ± SEM (n = 5 birds per each treatment).

Sperm Parameters: Mitochondrial activity (Rhodamine 123), apoptotic status (Annexin V/Propidium iodide), lipid peroxidation index (Malondialdehyde) and DNA Fragmentation (SDF) were assessed.

Abbreviations: PSO, pumpkin seed oil; SFO, sunflower oil; vitE, vitamin E.

^a-b^ Different letters within the same row show significant differences among the groups (*P* < 0.05).

1 Roosters received 6 types of treatments: 1) basal diet as control group (Control), 2) basal diet supplemented with 2% pumpkin seed oil as n-3 source of fatty acids (PSO group), 3) basal diet supplemented with 2% sunflower oil as n-6 source of fatty acids (SFO group), 4) basal diet supplemented with 200 mg/kg vitamin E (CTRL + vitE group), 5) basal diet supplemented and 2% pumpkin seed oil along with 200 mg/kg vitamin E (PSO + vitE group) and 6) basal diet supplemented with 2% sunflower oil along with 200 mg/kg vitamin E (SFO + vitE group).

Among the treatment groups, total motility and progressive motility were not different on d 1 and 20, however, the group 5 (PSO + vitE) had the greatest value on d 40 and 60 as compared with other groups (*P* < 0.05, Table 4, Figures 6 and 7). In terms of the velocity parameters, the VAP and STR were higher in groups 5 (PSO + vitE) on 40 and 60 d. Other kinematic parameters such as ALH, LIN, VCL and VSL were greatest in group 5 (PSO + vitE) as compared with other groups (Table 4, Figure 8).

MDA levels was lower in groups 5 (PSO + vitE) and 6 (SFO + vitE), respectively (Table 5, Figure 9). The mitochondrial activity (%) was not affected among the treatments on 40 and 60 days of the experiment (Table 5, Figure 10). DNA Fr+ (%) was not significantly affected on days of 20, 40, and 60. The group 5 (PSO + vitE) had numerically the lowest DNA Fr+ (%) at d 0 of the experiment (Table 5, Figure 11). DNA Fr^−^ (%) was lowest in group 6 (SFO+vitE) on day 40 the experiment. However, no significant difference (*P* > 0.05) was observed on 20 and 60 days of the experiment in terms of DNA Fr^−^ (%). The lowest sperm apoptosis (%) was observed by PSO + vitE group at different days of the experiment (Table 5, Figure 12).

### Hormonal Parameters

Testosterone level was not affected at 0 and 60 days of the experiment (*P >* 0.05). The concentration of LH and FSH changed on 0 d and 60 d among all the treatment groups (Table 6). The groups 2 (PSO) and 3 (SFO) had the least (*P* < 0.05) LH level among the treatments at 60 d. The concentration of FSH was not significantly affected by the different diets at day 60 (*P* > 0.05).

**Table 6:** Hormonal characteristics of roosters fed with fatty acid sources with or without vitamin E.

		Treatments 1							
Parameters	Days	1 (CTR group)	2 (PSO group)	3 (SFO group)	4 (CTR+vitE group)	5 (PSO+vitE group)	6 (SFO+vitE group)	SEM	*P*-value
Testosterone (nmol/L)									
	0	2.42	2.27	2.51	2.34	2.25	2.32	0.06	0.84
	60	2.5	2.2	2.19	2.32	2.22	2.35	0.1	0.96
LH (ng/L)									
	0	3.46 ^ab^	3.07 ^b^	3.25 ^ab^	3.83 ^a^	3.38 ^ab^	3.31 ^ab^	0.09	0.24
	60	2.72 ^bcd^	2.38 ^d^	2.27 ^d^	3.60 ^a^	3.34 ^ab^	3.12 ^abc^	0.13	0.01
FSH (IUI/L)									
	0	5.32 ^ab^	5.10 ^ab^	4.79 ^b^	5.53 ^a^	5.09 ^ab^	5.26 ^ab^	0.08	0.15
	60	5.36	5.18	5.48	5.14	5.28	5.28	0.06	0.68

Data are presented as means ± SEM (n = 5 birds per each treatment).

Hormonal Parameters: LH (Luteinizing hormone); FSH (Follicle-Stimulating Hormone).

^a-b^ Different letters within the same row show significant differences among the groups (*P* < 0.05).

Abbreviations: PSO, pumpkin seed oil; SFO, sunflower oil; vitE, vitamin E.

1 Roosters received 6 types of treatments: 1) basal diet as control group (Control), 2) basal diet supplemented with 2% pumpkin seed oil as n-3 source of fatty acids (PSO group), 3) basal diet supplemented with 2% sunflower oil as n-6 source of fatty acids (SFO group), 4) basal diet supplemented with 200 mg/kg vitamin E (CTRL + vitE group), 5) basal diet supplemented and 2% pumpkin seed oil along with 200 mg/kg vitamin E (PSO + vitE group) and 6) basal diet supplemented with 2% sunflower oil along with 200 mg/kg vitamin E (SFO + vitE group).

## DISCUSSION

Feeding strategies based on physiological mechanisms can change the physiological conditions of aged roosters and lead to better fertility. In these nutritional strategies, nutrients can be manipulated to improve fertility. Nowadays, lipid sources and unsaturated fatty acids have been shown to be the most important influencing factors and several cellular and molecular mechanisms have been discovered for this effect (Akhlaghi et al., 2014; Shariatmadari et al., 2020).

Fatty acids have been reported to increase the production of sex hormones, especially testosterone (Esmaeili et al., 2014). Fat sources are also part of the sperm plasma membrane and play a crucial role in sperm fertility. Therefore, in the present study, fatty acids were used as a nutrient to improve the fertility of older roosters. Given that many issues related to the use of fatty acid sources remain unknown, in this study the sources of omega-3 and omega-6 fatty acids were investigated. Due to the need of older roosters for this feeding treatment, this study was performed on older roosters (45 wk and older) whose reproductive performance are declining. This is the first study to evaluate the different effects of omega-3 omega-6 fatty acids sources with or without vitamin E on reproductive function as well as physiological parameters of reproduction in roosters.

It should be noted that in such studies, when a fat source is used in the diet, an antioxidant must be used, which prevents the peroxidation of fat sources. These antioxidants are also able to accumulate activated oxygen and then neutralize it inside and outside the body's cells (Cerolini et al., 2005). In this study, vitamin E antioxidant was used to protect the fat sources. In this study, parameters such as semen volume, sperm concentration, and sperm morphology were not affected by any of the experimental diets. It seems that due to the age of the roosters during the experimental period, these indicators did not improve.

In fact, it seems that the roosters were aging during the experimental period, and this prevented the improvement of these indices in the sperm. It was thought that since fatty acids could support spermatogenesis, we could see a better increase in parameters such as semen volume, sperm concentration, and morphology, which was not possible. These findings suggest that a decrease in sperm concentration in older birds cannot be reversed by polyunsaturated fatty acids (Akhlaghi et al., 2014). Other parameters including motility, viability, and plasma membrane integrity of sperm improved significantly after supplementation by fatty acid sources (omega-3) which were consistent with studies done on pigs, rams, bulls, and young roosters in diets with omega-3 and omega-6 (Feng et al., 2015; Masoudi et al., 2017; Qi et al., 2019). Thus far, different and contradictory results have been published regarding the effects of omega-3 fatty acid sources on reproductive indices in different species, which seems to be the reason for these differences and contradictions in their effects. It could be related to the age, breed, basic diet and breeding conditions of the animal, which affects the efficiency of omega-3 diet on the reproductive output (Bongalhardo et al., 2009). Improvement in some sperm-related indices and reproductive performance of roosters in this study under the influence of fatty acid sources are consistent with the study on Jing Hong rooster (Feng et al., 2015).

Avian sperm membrane lipids, like other species, have been reported to contain large amount of PUFAs. The unique property of avian sperm is the presence of small amounts of omega-3 and significant amounts of omega-6 (the ratio of omega-3 to omega-6 is 0.04 to 0.2 depending on the species). Therefore, it seems that the addition of omega-3 fatty acids through the diet has led to such an improvement in the reproductive parameters of roosters. It has generally been shown that the ratio of omega-3 to omega-6 is not high in the diets of birds, especially chickens and roosters, and supplementing the diet with omega-3 increases necessary fatty acids in sperm, which ultimately leads to higher motility and longer sperm survival rate which is clear in the present study. Although supplementation of PUFAs alters sperm fatty acid composition and may improve membrane flexibility, PUFAs are subject to the oxidation easily (Esmaeili et al., 2015; Alagawany et al., 2019). Therefore, it seems necessary to enrich diets containing unsaturated fatty acids with an antioxidant that can fight free radicals. In this study, diets with vitamin E showed that the amount of MDA was lower as an indicator of membrane lipid peroxidation than diets without vitamin E, which confirms the use of unsaturated fatty acids with several dual bonding without protection or without antioxidants can lead to cell damage in sperm, which increases the sensitivity to lipid oxidation. It is also recommended that the oxidation of fatty acids by several double bonds can be prevented by enzymatic and non-enzymatic antioxidants (Noei Razliqi et al., 2015). The reason for choosing vitamin E in this study was that vitamin E is one of the major antioxidants in sperm that has been widely identified in sperm membranes and it protects the sperm cell from oxidative damage (Benedetti et al., 2012). The results of this study confirm that maintaining the antioxidant status of sperm membranes by using antioxidant vitamin E increases sperm motility and survival. In addition, the findings of this study show that supplementing the diet with vitamin E prevented the occurrence or progression of sperm lipid peroxidation. On the other hand, adding vitamin E to the diet has been reported to have a positive effect on total motility and progressive motility of rooster sperm (Amini et al., 2015).

In the current study, the effects of fatty acid sources on hormones LH, FSH, and testosterone were also studied. Despite the fact that LH and FSH were not affected by the treatments, but the end product which is testosterone, was affected by the fatty acid sources. LH forces interstitial cells in the testes to produce testosterone, and FSH plays a critical role in spermatogenesis (Chimento et al., 2014). Our findings are inconsistent with the results of Feng et al. (2015), who reported that diets supplemented with PUFAs increase the GnRH pulse frequency in young roosters. There are possible reasons that why we did not see the change in LH and FSH. Because for the LH and FSH there are several fluctuations in amplitude and frequency of the hormone, we may have not selected the right time of blood sampling during the day. We sampled the blood at a specified time of day (10 to 11 AM) and the fluctuation of hormones in all the roosters may not have the same trend. In addition, they reported that LH, FSH, and testosterone increased in young broilers fed with omega-3 as fatty acid sources. Even though LH and FSH were not affected in our study by PUFAs, the end product of the pituitary-hypothalamic axis (testosterone) was increased, which is consistent with the study reported above. It is not clear why LH and FSH were not affected, but it is possible that the age of roosters and the time of blood sampling during the day had independent effects on hormonal outcomes.

## CONCLUSIONS

This is the first study to evaluate PSO as omega-3 fatty acid with vitamin E on reproductive functions of roosters. At 45 wk of age, when roosters experience reduced reproductive function, treating them with nutrient-enriched diets (especially omega-3 source) will improve their sexual conditions. Supplementation of pumpkin oil and vitamin E improved some semen quality parameters and reproductive performances in this study. It could be concluded that vitamin E as an antioxidant could remarkably improve the effects of omega-3 on sperm characteristics, seminal MDA, and sexual hormones levels in aged breeder roosters. Other aspects of pumpkin oil application in roosters’ diets such as effect on testicular structure needs special attention in the future studies. Future studies on the combination of different omega-3 and omega-6 fatty acid sources are also recommended.
